# Psychosocial impact of COVID‐19 for general workers

**DOI:** 10.1002/1348-9585.12132

**Published:** 2020-06-01

**Authors:** Akihito Shimazu, Akinori Nakata, Tomohisa Nagata, Yutaka Arakawa, Sachiko Kuroda, Nobuyuki Inamizu, Isamu Yamamoto

**Affiliations:** ^1^ Department of Policy Management Keio University Kanagawa Japan; ^2^ Department of Epidemiology and Social Medicine Graduate School of Public Health International University of Health and Welfare Tokyo Japan; ^3^ Institute of Industrial Ecological Sciences University of Occupational and Environmental Health, Japan Fukuoka Japan; ^4^ Graduate School of Information Science and Electrical Engineering Kyushu University Fukuoka Japan; ^5^ Faculty of Education and Integrated Arts and Sciences Waseda University Tokyo Japan; ^6^ Graduate School of Economics The University of Tokyo Tokyo Japan; ^7^ Faculty of Business and Commerce Keio University Tokyo Japan

**Keywords:** COVID‐19, general workers, mental health, psychosocial factors

We read with keen interest the recent editorial by Koh and Goh.^1^ We learned five insights proposed by authors that had been gained from the previous coronavirus outbreaks (ie severe acute respiratory syndrome [SARS], Middle East respiratory syndrome [MERS]). These insights mainly focused on infection control among occupational groups with high risk of infection and on psychosocial problems especially among healthcare workers. In addition to their insights, we propose a couple of other issues from viewpoint of psychosocial factors among general workers, more specifically, a variety of workers whose risk is low to moderate likelihood of exposure to the pathogen. Although risk of infection is reported to change according to occupational groups and considerably change even in nonhealthcare workers,^2^ social and psychological problems among those with low to moderate risks of infection are poorly understood by scientific community.

Many countries including Japan issued an emergency declaration. In this situation, business continuity is required in areas such as (a) maintenance of medical system; (b) protecting those who need help; (c) ensuring stable people's lives; and (d) maintaining social stability. Workers involved in these areas face moderate to high risks of becoming infected. They also face the increased demands with limited workforce, which can lead to overwork. Prolonged overwork reduces the opportunity for recovery from stress and can lead to an increased risk for overwork‐related disorders/deaths. In addition, extended working hours reduce time for off‐job activities such as relationship with family and friends, and general well‐being.

In contrast with overwork, serious deterioration of economy could have a negative impact on well‐being of workers. Authorities around the world are asking—and sometimes demanding—that citizens avoid public spaces, cancel mass gathering events, and simultaneous closure of stores. These strategies can reduce the risks of becoming infected. However, according to the ILO, almost 25 million jobs could be lost worldwide as a result of COVID‐19 pandemic.^3^ This economic and labor crisis might cause income‐ and employment‐related stress among workers, resulting in poorer workers' well‐being. A survey in Taiwan showed that respondents with considerable economic impacts of SARS had a significantly higher depressive level.^4^

Work from home will help business keep operating while workers stay safe. While work from home can avoid public transport and crowded places, lower the risks of becoming infected, and increase perceived autonomy, there are some drawbacks including social and professional isolation. A meta‐analysis showed that telecommuters' relationships with colleagues suffered if they worked remotely more than 2.5 days each week.^5^ Again, a survey in Taiwan revealed that respondents who had been kept isolated during SARS outbreak had a significantly higher depressive level.^4^ Along with social isolation, blurring of boundaries between work and personal life is a significant challenge for telecommuters and employers. A survey showed that telecommuters work an extra four hours per week compared with their counterparts in the office,^6^ suggesting that work interferes with personal life. On the other hand, family obligations such as caring for one's child(ren) can easily intrude into work hours especially for workers rearing school children. Given the increased number of infected people, the burden on workers who care for infected family members at home should be noted. We should also take into account those who do not have the freedom of being able to work from home because of business continuity. They face moderate to high risks of becoming infected and are worried about the continued risk of infection. Psychological care should be provided to them.

There are several suggestions of immediate strategies to protect mental health from psychological (eg coping, relaxation), physiological (eg sleep, nutrition), behavioral (eg physical activities), and social (eg communication) aspects. In addition, longer term strategies should be considered to promote psychosocial well‐being, including those based on positive mechanistically based components.^7^ For instance, problem‐focused coping can be helpful to maintain personal control over the situation, and the creation of positive (even minor) events in daily life provides momentary respite from chronic stress. Altruism and prosocial behaviors can increase the opportunity to elicit social support from others.^6^ Appropriate interventions should be developed at individual, workplace, and society levels with short‐ and longer‐term perspectives. We hope our proposal can help policy makers and occupational health professionals support health and well‐being of more diverse workers of during/after a pandemic.

## DISCLOSURE

*Approval of the research protocol*: N/A; *Informed consent*: N/A; *Registry and the registration no. of the study/trial*: N/A; *Animal studies*: N/A; *Conflict of interest*: We declare no competing interests.

## AUTHOR CONTRIBUTIONS

A Shimazu drafted the manuscript. A Nakata, T Nagata, Y Arakawa, S Kuroda, N Inamizu, and I Yamamoto provided critical revision of the manuscript for important intellectual content. All authors read and approved the final manuscript.
